# A Sterol-Regulatory Element Binding Protein Is Required for Cell Polarity, Hypoxia Adaptation, Azole Drug Resistance, and Virulence in *Aspergillus fumigatus*

**DOI:** 10.1371/journal.ppat.1000200

**Published:** 2008-11-07

**Authors:** Sven D. Willger, Srisombat Puttikamonkul, Kwang-Hyung Kim, James B. Burritt, Nora Grahl, Laurel J. Metzler, Robert Barbuch, Martin Bard, Christopher B. Lawrence, Robert A. Cramer

**Affiliations:** 1 Department of Veterinary Molecular Biology, Montana State University, Bozeman, Montana, United States of America; 2 Virginia Bioinformatics Institute, Virginia Polytechnic Institute and State University, Blacksburg, Virginia, United States of America; 3 Department of Microbiology, Montana State University, Bozeman, Montana, United States of America; 4 Department of Biology, Indiana University-Purdue University, Indianapolis, Indiana, United States of America; David Geffen School of Medicine at University of California Los Angeles, United States of America

## Abstract

At the site of microbial infections, the significant influx of immune effector cells and the necrosis of tissue by the invading pathogen generate hypoxic microenvironments in which both the pathogen and host cells must survive. Currently, whether hypoxia adaptation is an important virulence attribute of opportunistic pathogenic molds is unknown. Here we report the characterization of a sterol-regulatory element binding protein, SrbA, in the opportunistic pathogenic mold, *Aspergillus fumigatus*. Loss of SrbA results in a mutant strain of the fungus that is incapable of growth in a hypoxic environment and consequently incapable of causing disease in two distinct murine models of invasive pulmonary aspergillosis (IPA). Transcriptional profiling revealed 87 genes that are affected by loss of SrbA function. Annotation of these genes implicated SrbA in maintaining sterol biosynthesis and hyphal morphology. Further examination of the SrbA null mutant consequently revealed that SrbA plays a critical role in ergosterol biosynthesis, resistance to the azole class of antifungal drugs, and in maintenance of cell polarity in *A. fumigatus*. Significantly, the SrbA null mutant was highly susceptible to fluconazole and voriconazole. Thus, these findings present a new function of SREBP proteins in filamentous fungi, and demonstrate for the first time that hypoxia adaptation is likely an important virulence attribute of pathogenic molds.

## Author Summary

The incidence of potentially lethal infections caused by normally benign molds has increased tremendously over the last two decades. One disease in particular, invasive pulmonary aspergillosis (IPA), caused by the common mold *Aspergillus fumigatus*, has become the leading cause of death due to invasive mycoses. Currently, we have a limited understanding of how this opportunistic pathogen causes disease in immunocompromised patients. In this study, we discover a previously unexplored mechanism required by this mold to cause disease, hypoxia (low oxygen) adaptation. We report that hypoxia adaptation in *A. fumigatus* is mediated in part by a highly conserved transcription factor, SrbA, a protein in the sterol regulatory element binding protein family. A null mutant of SrbA was unable to grow in hypoxia, displayed increased susceptibility to the azole class of antifungal drugs, and was avirulent in two distinct murine models of IPA. Importantly, we report the discovery of a novel function of SrbA in molds related to maintenance of cell polarity. The finding that SrbA regulates resistance to the azole class of antifungal drugs presents an opportunity to uncover new mechanisms of antifungal drug resistance in *A. fumigatus*.

## Introduction

*Aspergillus fumigatus* is a normally benign saprophytic fungus that may cause an often lethal invasive disease in immunocompromised patients, invasive pulmonary aspergillosis (IPA) [1],[2]. Interestingly, while IPA can be caused by several *Aspergillus* species, the majority of IPA cases are caused by *A. fumigatus*. This may suggest that *A. fumigatus* contains unique attributes that allow it to cause disease [3]. Yet, the mechanisms utilized by *A. fumigatus* to survive and cause disease in immunocompromised hosts are not fully understood [4]. During infection, *A. fumigatus* causes significant damage to host tissue through invasive growth by hyphae and subsequent recruitment of immune effector cells. Thus, infection generates significant inflammation and necrosis in lung tissue that can be visualized by histopathology. These pathologic lesions also likely represent areas of poor oxygen availability to the pathogen and host.

At sites of *Aspergillus* infection, direct measurements of oxygen tension have not been recorded, however, it is well established that sites of inflammation contain significantly low levels of oxygen (hypoxia) [5]–[7]. Moreover, low oxygen tension has been observed in many compartments of inflamed as well as normal tissues [5]–[7]. In inflamed tissues, the blood supply is often interrupted because the vessels are congested with phagocytes [8],[9]. Indeed, immune effector cells such as neutrophils often function effectively in severely hypoxic microenvironments and have evolved distinct mechanisms to deal with the absence of oxygen that are dependent upon the transcription factor hypoxia inducible factor (HIF) 1.

HIF1 is a heterodimeric transcription factor that consists of a constitutively expressed HIF1β subunit and an oxygen-tension-regulated HIF1α subunit. [10]. Increased HIF1α protein stability and activity of the HIF1 complex, in turn, regulate the transcription of many hypoxia-responsive genes, including those encoding many glycolytic enzymes, erythropoietin, adrenomedullin, and growth factors [11],[12]. Genetic evidence of the importance of hypoxic environments in the regulation of immune responses was recently provided by a study of neutrophil-mediated lung inflammation [13]. Thus, since immune cells of the host have evolved mechanisms to function in hypoxia, it follows that invasive fungal pathogens like *A. fumigatus* are likely subjected to hypoxia during fungal pathogenesis.

While hypoxic adaptation has not been studied in the context of *A. fumigatus* pathogenesis, circumstantial evidence suggests that hypoxia plays a key role in the pathophysiology of IPA. For example, it has been postulated that the low rate of *Aspergillus* recovery from clinical specimens is due to adaptation by the fungus to hypoxic microenvironments found at sites of infection [14],[15]. Furthermore, there are often significant differences in the *in vivo* and *in vitro* test results of antifungal drug efficacies. These differences have been postulated to be linked to the occurrence of hypoxia *in vivo* as demonstrated by recent *in vitro* antifungal drug efficacy tests conducted in hypoxia [16],[17]. Consequently, it seems probable that pathogenic molds such as *A. fumigatus* must possess mechanisms to adapt to hypoxic microenvironments found *in vivo* during infection.

In fact, switching from aerobic respiration to various forms of anaerobic respiration to deal with low oxygen levels has been implicated as an important virulence attribute in several prokaryotic pathogens [18],[19]. However, in eukaryotic pathogens, mechanisms of how these organisms respond and adapt to hypoxia are largely unknown. Most of our knowledge on how fungi respond to hypoxia comes from studies in the model yeast *Saccharomyces cerevisiae*. Under aerobic conditions, heme biosynthesis activates the transcriptional regulator Hap1p [20]. Hap1p induces genes involved in respiration and oxidative stress-responses, but also activates the transcriptional repressors Rox1p and Mot3p, that down-regulate genes required for hypoxia adaptation [21]. However, in hypoxic conditions, Rox1p and Mot3p are expressed and this leads to transcriptional induction of genes involved in hypoxia adaptation [22]. Thus, hypoxic gene expression in yeast requires transcription factors that utilize Rox1p-binding sequences, low oxygen-response elements (LORE), and other regulatory elements within promoters [23],[24]. Since *S. cerevisiae* is a facultative anaerobe, it is not surprising that homologs of these key hypoxia gene regulators have not been found in obligate aerobic filamentous fungi such as *A. fumigatus*.

Recently, a novel mechanism of hypoxia adaptation mediated by a highly conserved family of transcription factors, sterol regulatory element-binding proteins (SREBPs), was characterized in fission yeast, *Schizosaccharomyces pombe* [25]. SREBPs are a unique family of membrane bound transcription factors first identified in mammals as regulators of cholesterol and lipid metabolism [26]–[30]. Hughes et al. [25] proposed a model in *S. pombe* where SREBP (Sre1) and a sterol cleavage activating protein (SCAP, Scp1) monitor-oxygen dependent sterol synthesis as an indirect measure of oxygen supply. Importantly, Sre1 was found to be required for adaptation to hypoxia and regulated approximately 68% of the genes transcriptionally induced greater than 2-fold in response to anaerobic conditions [31].

Orthologs of Sre1 and Scp1 were recently identified and characterized in the human fungal pathogenic yeast, *Cryptococcus neoformans* [32],[33]. As in fission yeast, the SREBP pathway mediated by Sre1 and Scp1 in *C. neoformans* was crucial for adaptation to hypoxia and sterol biosynthesis. Importantly, these mutants also failed to proliferate in host tissue, failed to cause fatal meningoencephalitis, and displayed hypersensitivity to the azole class of antifungal drugs [32],[33]. In the yeast *S. cerevisiae and Candida albicans*, orthologs of SREBPs do not appear to exist. However, two similar genes, *Upc2 and Ecm22*, appear to serve similar functions as SREBPs. Conserved functions of these genes include their involvement in the ability of yeast to grow in hypoxia as well as regulation of sterol biosynthesis and resistance to antifungal drugs [34]–[40]. Taken together, these observations demonstrate an important link between sterol biosynthesis, hypoxia adaptation, azole drug resistance, and the virulence of pathogenic yeasts.

In this study, we report the identification and first characterization of a Sre1 homolog, SrbA, in an opportunistic pathogenic mold, *A. fumigatus*. Our results suggest that while certain aspects of SREBP function are conserved in yeast and filamentous fungi, significant differences exist that are unique to molds. Thus, our results further expand the spectrum of important functions mediated by SREBPs in eukaryotes, emphasize the importance of this pathway in human fungal pathogenesis, and suggest possible clinical significance of SREBPs related to antifungal drug efficacy.

## Results

### Identification of *srbA* in *Aspergillus fumigatus*

In order to determine if hypoxia adaptation is an important virulence attribute of filamentous fungi, we first conducted transcriptional profiling experiments using a long-oligo *A. fumigatus* microarray (version 3.0) of wild type *A. fumigatus* grown under hypoxic (1% O_2_) conditions compared to fungus grown under normal conditions (∼21% O_2_). Analysis of this data revealed ten putative transcription factors that were transcriptionally induced more than 2-fold in response to hypoxia and thus could possibly act as a regulators of genes required for hypoxic adaptation in *A. fumigatus* (Willger and Cramer, unpublished data). Further bioinformatic analyses of these genes revealed that only one, AFUA_2g01260 (induced 5.02 fold in response to hypoxia), had similarity with a functionally characterized protein, Sre1 from *S. pombe* [25]. Sre1 shares similarity with mammalian SREBP proteins that regulate lipid and cholesterol homeostasis (reviewed in [29],[30]). In addition, a Sre1 homolog has also recently been described in the human pathogenic yeast, *C. neoformans* as a regulator of hypoxia adaptation and fungal virulence [32],[33]. AFUA_2g01260 contains 988 amino acid residues, which displayed low sequence percent identity with Sre1 from *S. pombe* (∼13%) and *C. neoformans* (∼10%). However, like Sre1 in both yeasts, the amino terminus (amino acids 1–425) of AFUA_2g01260 contains a basic helix-loop-helix (bHLH) leucine zipper DNA binding domain. In addition, AFUA_2g01260 is predicted to contain at least one, and likely two, transmembrane domains. The carboxyl terminus of AFUA_2g01260 is also predicted to contain a conserved domain of unknown function (DUF2014) that is found in other SREBP homologs. Consequently, these results suggest that AFUA_2g01260 is likely the SREBP homolog in *A. fumigatus*, and we consequently named this gene *srbA* (*sreA* is already in use in *A. nidulans* for an unrelated gene). Additional BLAST analyses revealed that SrbA is highly conserved amongst the filamentous fungi with putative orthologs found in plant pathogens such as *Magnaporthe grisea and Alternaria brassicicola* and saprophytic molds such as *Neurospora crassa* and *Aspergillus nidulans*.

### SrbA is required for hypoxia adaptation in *Aspergillus fumigatus*

**Figure 1:**
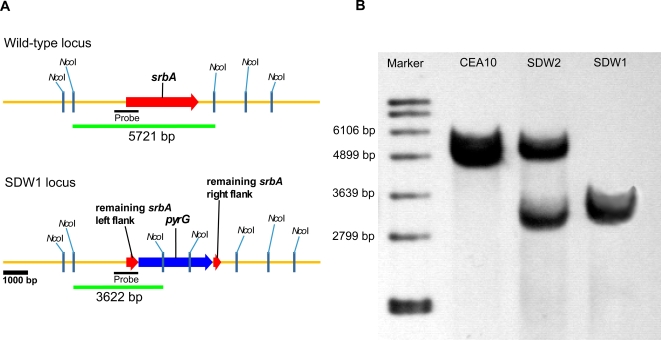
Generation and confirmation of a SrbA null mutant in *Aspergillus fumigatus*. (A) Schematic of wild type (CEA10) and SDW1 (SrbA null mutant) genomic loci. (B) Southern blot analysis of wild type, SDW1, and SDW2 strains. Genomic DNA from the respective strains was isolated and digested overnight with *Nco*I. An approximate 1 kb genomic region of the SrbA locus was utilized as a probe. The expected hybridization patterns and sizes were observed for the wild type CEA10 (5721 bp) and SrbA mutant (SDW1) (3622 bp) strains. In addition, confirmation of ectopic reconstitution of the SrbA null mutant was confirmed by the presence of the wild type *srbA* locus hybridization signal and persistence of the SrbA null mutant locus (strain SDW2).

**Figure 2:**
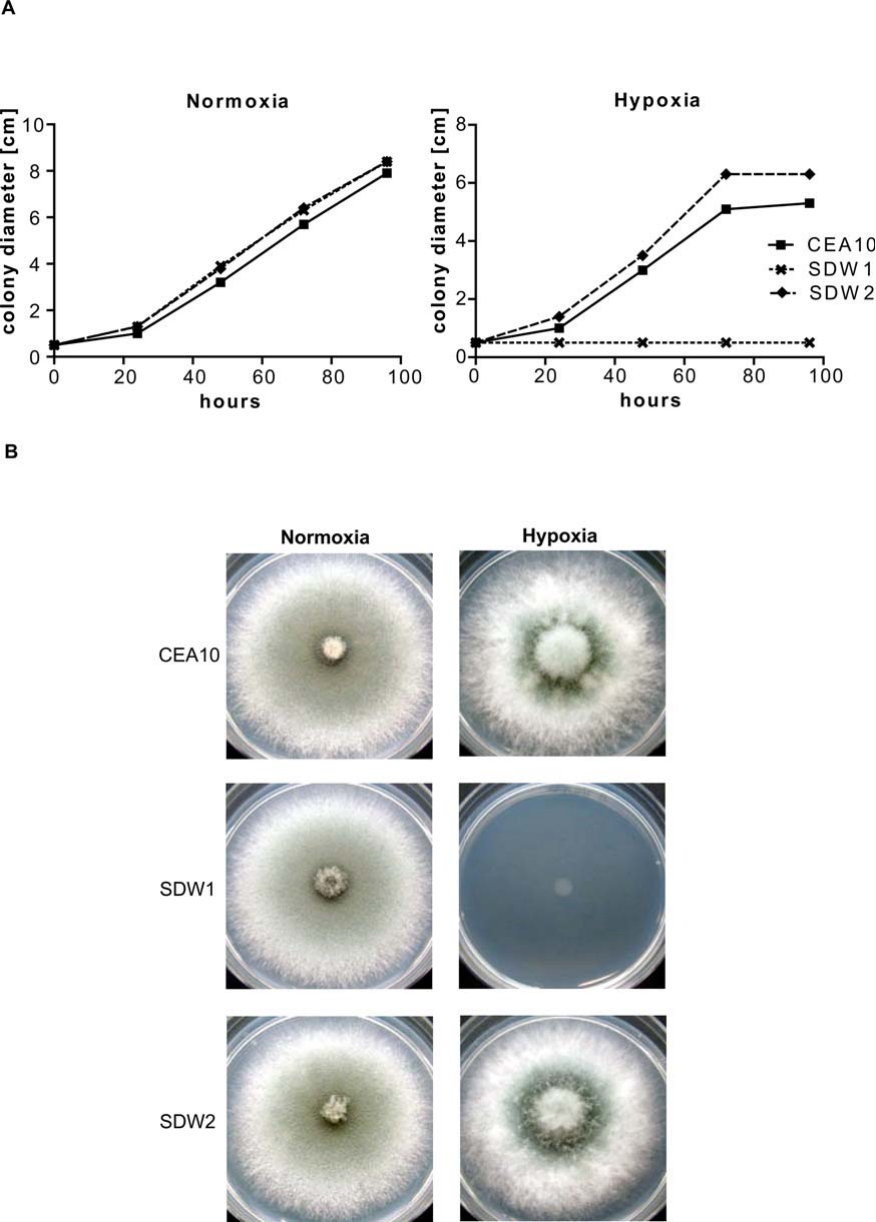
SrbA is required for hyphal growth under hypoxic conditions. 1×10^6^ conidia of CEA10, SDW1 = *ΔsrbA*, SDW2 = *ΔsrbA+srbA* were plated on GMM plates and incubated at 37°C under normoxic and hypoxic conditions. (A) The diameter of the colony was measured over 96 h every 24 h. Under normoxic conditions no significant difference in growth speed and colony size or morphology could be observed (P>0.01). (B) Under hypoxic conditions the wild type CEA10 and the reconstituted strain SDW2 showed comparable growth (P>0.01) but the mutant strain SDW1 did not demonstrate any detectable growth. Error bars represent standard error from the triplicate experiments.

To determine whether SrbA is involved in hypoxia adaptation and fungal virulence in filamentous fungi, we generated a null mutant of the gene encoding SrbA by replacement of the *srbA* coding sequence in *A. fumigatus* strain CEA17 with the auxotrophic marker *pyrG* from *A. parasiticus* as previously described [41],[42] (Figure 1). The resulting *ΔsrbA* strain was named SDW1. Ectopic re-introduction of the wild type *srbA* allele into SDW1 (resulting in strain SDW2) allowed us to attribute all resulting phenotypes specifically to the absence of *srbA* in SDW1. All strains were rigorously confirmed with Southern blot (Figure 1) and PCR analyses (data not shown). The re-introduced *srbA* allele in SDW2 displayed similar mRNA abundance in response to hypoxia as the *srbA* allele in the wild type strain (data not shown). SDW1 and SDW2 both displayed normal hyphal growth rates compared to the wild type strain CEA10 in normoxic conditions on glucose minimal medium (GMM) (Figure 2A) (P>0.01). However, no hyphal growth of SDW1 was observed in hypoxic (1% O_2_, 5% CO_2_, 94% N_2_) conditions whereas wild type strain CEA10 and reconstituted strain SDW2 grew at a normal rate with visual phenotypic differences in colony color and conidiation compared to growth in normoxia (Figure 2A and 2B). In hypoxia, the wild type strains displayed increased aerial hyphae, decreased conidia production, and consequently exhibited a fluffy colony morphology (Figure 2B). After 96 hours of incubation in hypoxia, SDW1 continued to display undetectable growth. However, upon transfer back to normoxic conditions, wild type growth rate was restored (data not shown). Addition of exogenous ergosterol or lanosterol did not rescue the SDW1 growth defect or alter wild type growth morphology in hypoxia (data not shown). These results indicate that *A. fumigatus* can rapidly adapt to hypoxic microenvironments, and that SrbA in *A. fumigatus* is involved in mediating this response by an undefined mechanism.

### Transcriptional profiling of SDW1 in response to hypoxia

Given the dramatic phenotype observed in strain SDW1 in hypoxia and the sequence annotation that SrbA likely functions as a transcription factor, we next sought to determine which genes are regulated by SrbA under hypoxic conditions. Microarray experiments comparing the transcriptional profiles of wild type strain CEA10 and SDW1 exposed to hypoxia for 24 hours revealed 87 significant genes possibly regulated by SrbA (Table 1). Several genes previously shown to be involved in ergosterol biosynthesis in fungi were found to be transcriptionally repressed in the absence of SrbA including *ERG25*, *ERG24*, and *ERG3* (Table 1). Interestingly, besides *srbA* itself, the gene with the highest fold difference in expression in SDW1 is a non-ribosomal peptide synthetase, AFUA_1g10380 (NRPS1 or *pes1*) [43],[44]. In addition, a significant number of genes involved in cell wall biosynthesis or homeostasis were observed to be repressed in SDW1 compared to wild type. These included genes known to be involved in cell wall biosynthesis such as alpha-galactosidase, alpha-glucosidase B, and genes involved in cell wall homeostasis such as chitinase. However, no obvious defects in cell wall biosynthesis were observed in the mutant strain, and thus the transcriptional profiling results are likely indirect effects of the altered cell polarity of the mutant (discussed below). Genes encoding several transporters were also found to be regulated by SrbA. Overall, these results suggest some similarities, such as with regard to ergosterol biosynthesis, with genes regulated by SREBPs in *S. pombe* and *C. neoformans*. However, the overall set of genes putatively regulated by SrbA in *A. fumigatus* is significantly different from data obtained from Sre1 mutants in the yeast *S. pombe and C. neoformans*. Consequently, these results strongly suggest that SrbA plays a distinct role in filamentous fungal biology. These results subsequently directed experiments to further characterize the role of SrbA in *A. fumigatus* biology.

**Table 1:** Genes with higher expression in wild type than in the *srbA* null mutant in hypoxia.

**TRANSPORTERS**		
AFUA_4g01560	MFS myo-inositol transporter, putative	15.77
AFUA_1g10390	ABC multidrug transporter, putative	15.64
AFUA_3g01670	MFS hexose transporter, putative	14.12
AFUA_2g09450	carboxylic acid transport protein	12.18
AFUA_3g14170	high-affinity hexose transporter	11.31
AFUA_5g06720	MFS sugar transporter, putative	11.04
AFUA_7g06120	transmembrane transporter, putative	10.80
AFUA_3g12720	sugar transporter-like protein	9.08
AFUA_1g13350	transporter, putative	7.65
**TRANSCRIPTION REGULATION**		
AFUA_2g01260	HLH transcription factor, putative (srbA)	38.59
AFUA_3g12910	MmcR, putative	10.49
AFUA_8g00200	CalO6, putative	8.05
AFUA_2g07900	APSES transcription factor (StuA), putative	7.79
AFUA_1g16590	C2H2 transcription factor (BrlA), putative	7.14
AFUA_8g05460	bZIP transcription factor, putative	6.09
**STEROL BIOSYNTHESIS**		
AFUA_8g02440	c-4 methyl sterol oxidase (ERG25)	15.79
AFUA_2g00320	sterol delta 5,6-desaturase (ERG3)	13.57
AFUA_1g03150	c-14 sterol reductase (ERG24)	11.98
**OXIDATIVE STRESS RESPONSE**		
AFUA_1g10380	nonribosomal peptide synthase (NRPS), putative	23.36
AFUA_7g05070	FAD dependent oxidoreductase, putative	13.68
AFUA_5g08830	HEX1	10.66
AFUA_4g08710	short chain dehydrogenase, putative	9.82
AFUA_3g02270	mycelial catalase Cat1	9.15
AFUA_4g14530	theta class glutathione S-transferase	8.45
AFUA_3g01580	GMC oxidoreductase	6.56
AFUA_1g03250	oxidoreductase, short chain dehydrogenase/reductase family, putative	6.25
**CELL WALL RELATED**		
AFUA_3g08110	cell wall protein, putative	21.29
AFUA_5g14740	fucose-specific lectin	15.00
AFUA_6g00430	IgE-binding protein	12.65
AFUA_5g00840	integral membrane protein	12.21
AFUA_5g03760	class III chitinase ChiA1	7.68
AFUA_4g09600	GPI anchored protein, putative	6.32
AFUA_2g05340	1,3-beta-glucanosyltransferase, putative	6.29
AFUA_1g05790	GPI anchored protein, putative	6.20
AFUA_5g07190	beta-glucosidase	6.04
**SECONDARY METABOLISM**		
AFUA_8g01220	arthrofactin synthetase B	8.38
AFUA_2g17600	polyketide synthetase PksP	6.40
**OTHER METABOLIC PROCESSES**		
AFUA_3g00810	cholestenol delta-isomerase, putative	18.48
AFUA_7g04930	alkaline serine protease (PR1), putative	13.09
AFUA_5g02130	alpha-galactosidase	12.92
AFUA_8g00190	cytochrome P450, putative	11.40
AFUA_1g16250	alpha-glucosidase B	9.95
AFUA_3g12960	cytochrome P450, putative	8.97
AFUA_3g07030	glutaminase A	8.24
AFUA_8g00620	dimethylallyl tryptophan synthase, putative	7.65
AFUA_4g09980	cytochrome P450 monooxygenase, putative	7.15
**OTHER AND UNKNOWN GENES**		
AFUA_6g03680	hypothetical protein	13.55
AFUA_3g07870	conserved hypothetical protein	13.23
AFUA_8g04380	conserved hypothetical protein	12.96
AFUA_8g00710	antimicrobial peptide, putative	12.92
AFUA_3g13110	hypothetical protein	12.88
AFUA_7g01930	ESDC	12.14
AFUA_4g08400	hypothetical protein	11.16
AFUA_6g01870	hypothetical protein	10.71
AFUA_4g14060	conserved hypothetical protein	10.60
AFUA_6g12180	conserved hypothetical protein	10.20
AFUA_6g13980	prenyltransferase, putative	10.04
AFUA_2g00500	conserved hypothetical protein	9.61
AFUA_8g04310	conserved hypothetical protein	9.26
AFUA_1g02290	conserved hypothetical protein	9.13
AFUA_7g05450	SUN domain protein (Uth1), putative	9.02
AFUA_2g15200	conserved hypothetical protein	8.86
AFUA_7g04870	hypothetical protein	8.71
AFUA_5g00700	hypothetical protein	8.69
AFUA_3g07730	hypothetical protein	8.65
AFUA_4g12700	hypothetical protein	8.60
AFUA_5g14920	hypothetical protein	8.59
AFUA_3g07340	hypothetical protein	8.53
AFUA_7g04120	DUF636 domain protein	8.23
AFUA_1g14340	metalloreductase, putative	8.01
AFUA_3g08210	hypothetical protein	7.95
AFUA_5g14680	hypothetical protein	7.80
AFUA_6g11890	dynamin GTPase, putative	7.72
AFUA_2g09030	secreted dipeptidyl peptidase	7.70
AFUA_4g13630	hypothetical protein	7.51
AFUA_2g09680	PB1 domain protein, putative	7.09
AFUA_7g04740	hypothetical protein	6.84
AFUA_3g12230	hypothetical protein	6.81
AFUA_5g02330	major allergen Asp F1	6.81
AFUA_6g14340	related to berberine bridge enzyme (imported)	6.64
AFUA_2g17550	yellowish-green 1	6.56
AFUA_8g04620	hypothetical protein	6.37
AFUA_4g14040	Hsp70 family protein	6.30
AFUA_5g14410	cysteine dioxygenase	6.30
AFUA_2g08820	hypothetical protein	6.24
AFUA_1g13610	SH3 domain protein	5.97
AFUA_4g14050	hypothetical protein	5.73

### SrbA mediates resistance to the azole class of antifungal drugs

**Figure 3:**
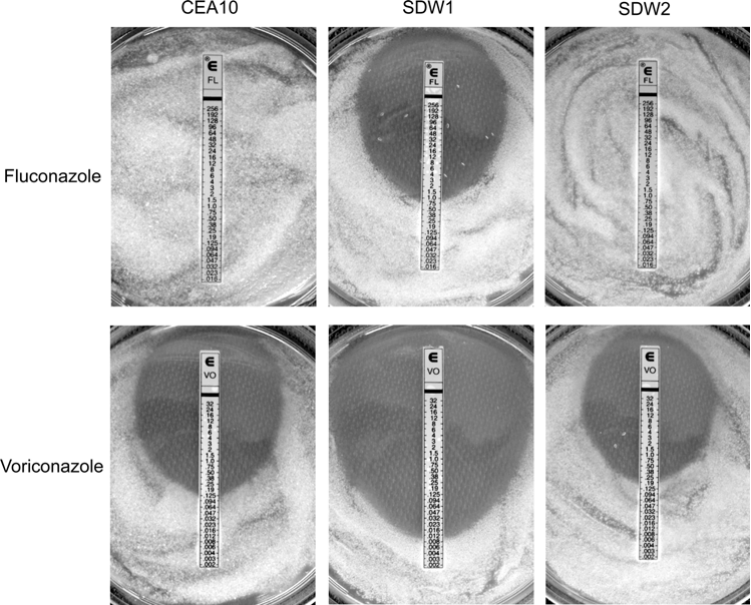
SrbA mediates resistance to Fluconazole (FL) and Voriconazole (VO) in *Aspergillus fumigatus*. A clear ellipse indicates the susceptibility to the respective drug. As expected, Fluconazole has no effect on CEA10 and SDW2, but in the absence of SrbA, SDW1 is highly susceptible to Fluconazole (MIC = 1.0 µg/ml). CEA10 and SDW2 are susceptible to Voriconazole (MIC for both = 0.125 µg/ml); however, SDW1 also displays increased susceptibility (MIC = 0.012 µg/ml) to this important antifungal agent. The numbers on the scale correspond to the Fluconazole and Voriconazole concentrations on the E-test strip (in micrograms per milliliter).

Given the number of ergosterol biosynthesis genes apparently regulated by SrbA, we next asked the question whether SrbA mediated resistance to the azole class of antifungal drugs that target ergosterol biosynthesis. In a screen for susceptibility to antifungal drugs using E-Test strips (AB Biodisk, kindly provided by Dr. Theodore White, Seattle Biomedical Research Institute) we found that SrbA is required for resistance to Fluconazole and Voriconazole, but not Amphotericin B or Caspofungin. All 3 strains showed equivalent minimal inhibitory concentrations (MIC) to Amphotericin B (0.25 µg/ml) and Caspofungin (0.125 µg/ml). The lack of effect of Caspofungin provides support for our hypothesis that the mutant is likely not directly affected in cell wall biosynthesis as possibly suggested by the transcriptional profiling data. However, while CEA10 and SDW2 showed resistance to Fluconazole as expected, SDW1 growth was inhibited at the surprisingly low MIC of 1 µg/ml (Figure 3). On the plates with Voriconazole we could observe that CEA10 and SDW2 were susceptible as expected (MIC of 0.125 µg/ml respectively). Similar to the results with Fluconazole, SDW1 was significantly more susceptible to Voriconazole and showed a MIC of only 0.012 µg/ml (Figure 3). These clinically significant results suggest that SrbA mediates resistance to the azole class of antifungal drugs by an undefined mechanism.

### SrbA is required for cell polarity and hyphal morphogenesis

**Figure 4:**
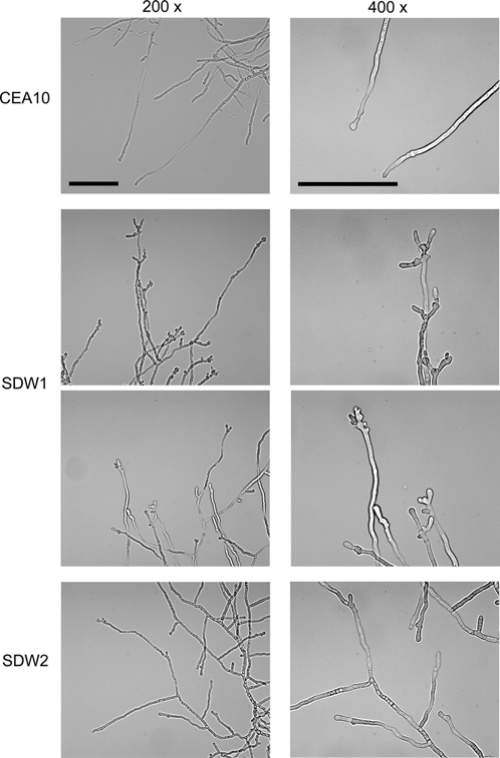
Hyphal morphology and growth of wild type strain CEA10 and SrbA null mutant SDW1. Strains were grown overnight on slides coated with GMM. Brightfield microscopy pictures of wild type CEA10 and SDW1 at 200-fold and 400-fold magnification. SDW1 showed abnormal hyphal formation and apparent cell polarity defect with multiple branches and unusual thick structures at the apical tips of the hyphae. Bars = 100 µm.

**Figure 5:**
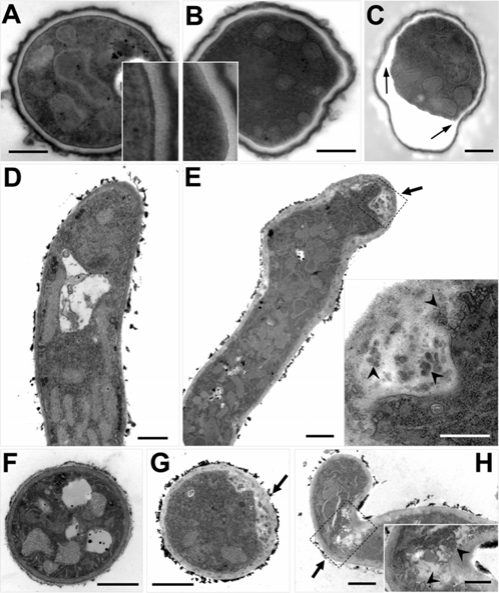
Abnormal cell wall-plasma membrane interface and hyphal morphology is evident in the absence of SrbA. (A–C) Transmission electron micrographs showing sections of conidia of wild type CEA10 (A) and SDW1 (B,C). Compared with the round wild type conidia having clear boundaries between plasma membrane and cell wall layers, most of the SDW1 conidia were distorted in shape and possessed faint, somewhat shriveled boundaries. Note that frequent “tearing” took place mainly at the cell wall – plasma membrane interface during microscopic examination of the SDW1 conidia (arrows). This phenotype was observed in over 80% of SDW1 conidia examined. Inset panels depict a 3× magnified view of the conidial cell wall region. Bars = 500 nm. (D–H). Transmission electron micrographs showing longitudinal and transverse hyphal sections of wild type CEA10 (D,F) and SDW1 (E,G,H). Close observation of the hyphal tips show phenotypic differences between wild type and SDW1. Abnormal cell wall – plasma membrane interfaces and apical swellings in SDW1 hyphae were frequently observed, while the wild type showed normal round-shaped apexes. With respect to cell wall morphology around the hyphal apex, SDW1 had an abnormally expanded cell wall (arrows) containing numerous electron dense objects (arrowheads), which likely resulted in hyphal tip bending (H). Inset panels depict a magnified view of the boxed region. Bars = 1 µm, except for the inset panels of E and H where they denote 500 nm.

**Figure 6:**
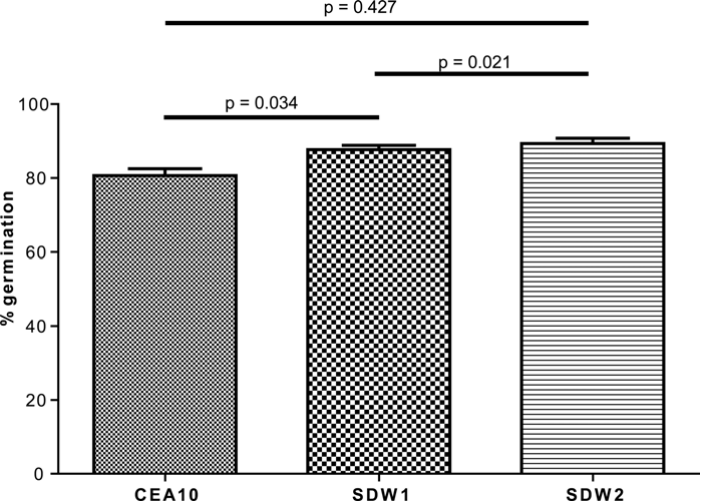
Conidia germination is not affected by loss of SrbA. Germination media was inoculated with approximately 10^6^ conidia/ml of the *A. fumigatus* strains CEA10, SDW1, and SDW2. After 7 hours the germination rate was determined by counting a total of 100 spores and noting the number of germinated spores. Three replicates were performed. No significant difference in germination was observed between CEA10, SDW1, and SDW2 (P>0.01).

Visual inspection of SDW1 colony morphology in standard laboratory conditions did not reveal any apparent morphological phenotypes (Figure 2B). However, our transcriptional profiling experiments suggested possible alterations in cell wall biosynthesis, a critical component of hyphal morphology and growth, in the absence of SrbA. Consequently, we performed a more in depth analysis of SDW1 morphology. First, we utilized light microscopy to examine the growing edges of SDW1 colonies in normoxia. We observed a significant defect in hyphal tip branching in SDW1 that is not apparent in strains CEA10 and SDW2 (Figure 4). SDW1 hyphal tips display hyper-branching and a “blunted” abnormal morphological phenotype (Figure 4). This phenotype suggests that SrbA is involved in maintaining cell polarity that directs hyphal growth. Interestingly, this phenotype does not appear to alter the growth rate of the colony, which was comparable to the wild type under normoxic conditions (Figure 2A). Next, we utilized transmission electron microscopy (TEM) to further examine the cell wall and morphology of conidia and hyphae of SDW1. Confirming our suspicions that the mutant was not directly affected in cell wall biosynthesis we observed no clear cell wall defects. However, a general thickening of the intracellular space between the cell wall and plasma membrane is observed in SDW1 conidia and hyphae compared with the wild type (Figure 5A and 5B and 5D and 5E). A striking phenotype was consequently observed in conidia from SDW1 that suggested a significant defect in the cell wall-plasma membrane interface occurs in the absence of SrbA (Figure 5A and 5B). This defect is apparently exacerbated by the electron beam, which causes a separation between the cell wall and plasma membrane in SDW1 conidia (Figure 5C). This phenotype was observed in over 80% of the SDW1 conidia examined. However, the size and density of the mutant conidia were comparable to the wild type strain as measured by flow cytometry (data not shown). Since a defect in the cell wall plasma membrane interface was suggested, we examined viability of the SDW1 conidia by monitoring germination. These experiments revealed that viability, as measured by conidia germination, was not significantly different between the wild type, SDW1 and SDW2 strains (Figure 6) (P>0.01). Similar cell wall-plasma membrane defects were observed in the SDW1 hyphae compared with the wild type hyphae (Figure 5D and 5E). Importantly, an accumulation of electron dense objects was observed in the SDW1 hyphae. We hypothesize that these objects may be vesicles of the Spitzenkörper, and their abnormal location in the SDW1 hyphae may cause the observed altered cell polarity (Figure 5E and 5H). This phenotype was observed in over 50% of the SDW1 hyphae examined and never observed in the wild type strain. These results suggest that SrbA is critical for maintaining the cell wall – plasma membrane interface, and that SrbA is critical for normal hyphal branching and cell polarity in filamentous fungi by an undefined mechanism.

### SrbA is required for normal sterol biosynthesis

**Figure 7:**
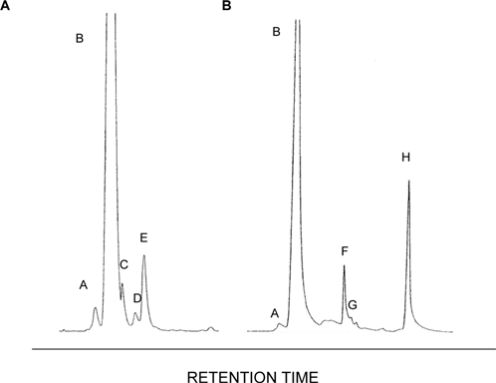
C4-demethylation is altered in the absence of SrbA. Representative GC-MS chromatograms of sterol extracts from wild type (A) and SDW1 (B). Key: A- ergosta-5,8,22-trien-3β-ol, B- ergosterol, C- ergosta-5,7,22,24(28)-tetraen-3β-ol, D- ergosta-5,7,24(28)-trien-3β-ol, E- 24-ethylcholesta-5,7,22-trien-3β-ol, F- 4-methylfecosterol, G- 4methylergosta-5,8,24(28)-trien-3β-ol, H- 4,4-demethylergosta-8,24(28)-dien-3β-ol. An accumulation of 4-methyl sterols is observed in the absence of SrbA, suggesting a blockage in enzymes involved in sterol C-4 demethylation. The ratio of C-4 methylated sterols to ergosterol in the absence of SrbA was 1.94 whereas no C-4 methylated sterols accumulated in the wild type.

Transcriptional profiling of SDW1 under hypoxia suggested that SrbA was involved in both early and late steps of the sterol biosynthesis pathway. In addition, the abnormal conidial and hyphal morphology observed via light microscopy and TEM micrographs in SDW1 also suggested possible alterations in sterol content in the absence of SrbA. Thus, we examined the sterol profile of the SrbA null mutant SDW1 by GC-MS and compared it with the wild type strain CEA10. The GC-MS profiles demonstrated a significant accumulation of 4-methyl sterols in the SrbA null mutant, SDW1, that was not observed in the wild type strain CEA10 (Figure 7). Interestingly, both strains possessed significant amounts of ergosterol (Figure 7). The ratio of C-4 methylated sterols to ergosterol in the absence of SrbA is 1.94 whereas no C-4 methylated sterols accumulated in the wild type. Specifically, the accumulation of 4-methylfecosterol and 4,4-dimethylergosta-8,24(28)-dien-3β-ol in the absence of SrbA suggests a blockage at ERG25 in the sterol biosynthesis pathway in SDW1. These alterations are supported by the transcriptional profiling data, which suggests transcriptional regulation of ERG25 by SrbA in *A. fumigatus* (Table 1). Consequently, these results suggest a blockage of C4 demethylation in the absence of SrbA in *A. fumigatus*. In addition, these results suggest that ergosterol can still be synthesized in the absence of SrbA in *A. fumigatus*.

### SrbA is required for fungal virulence in two distinct murine models

**Figure 8:**
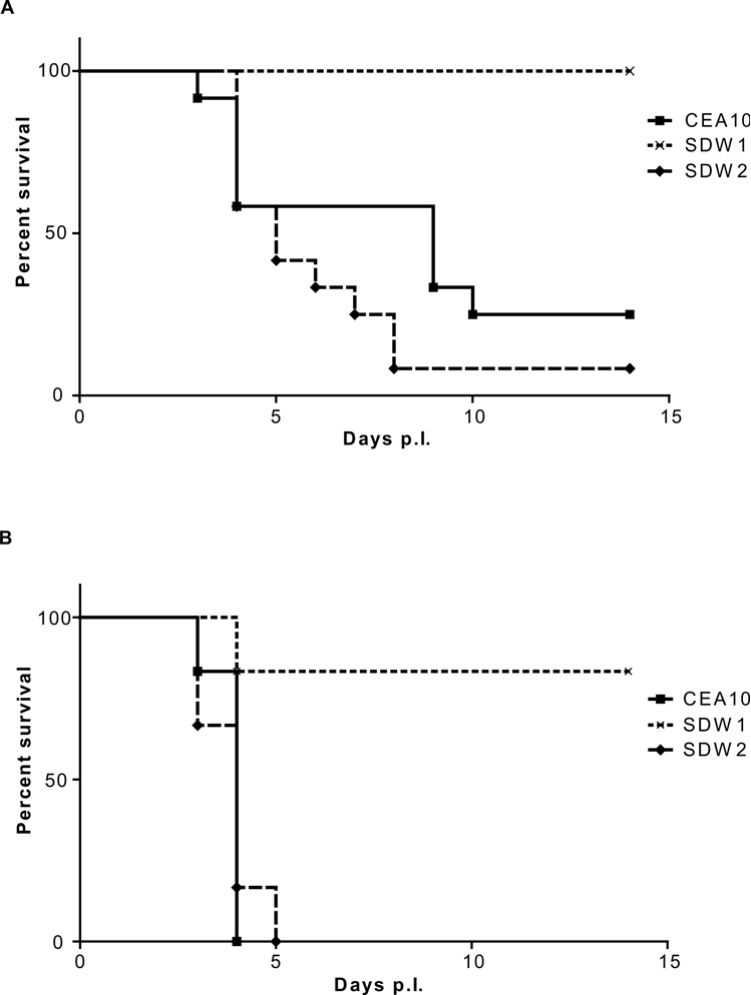
Role of SrbA in *Aspergillus fumigatus* virulence. (A) Outbred CD-1 mice (n = 12) were immunosuppressed by i.p. injection of cyclophosphamide (150 mg/kg) 2 days prior to infection and s.c. injection of Kenalog (40 mg/kg) 1 day prior to infection and injection of 150 mg/kg cyclophosphamide 3 days post-inoculation and 40 mg/kg Kenalog 6 days post-inoculation. Mice were inoculated intranasally with 10^6^ conidia in a volume of 40 µl of wild type CEA10, *ΔsrbA* mutant strain SDW1 and the *srbA* reconstituted strain SDW2. P value for comparison between SDW1 and wild type CEA10, P = 0.0002. (B) gp91*^phox−/−^* mice (n = 6) were challenged intratracheally with 10^6^ conidia in a volume of 40 µl of wild type CEA10, *ΔsrbA* mutant strain SDW1 and the *srbA* reconstituted strain SDW2. A log rank test was used for pair wise comparisons of survival levels among the strain groups. P value for comparison between SDW1 and wild type CEA10, P = 0.0054. SDW1 is significantly less virulent than the wild type CEA10 and the reconstituted strain SDW2 in both murine models. All animal experiments were repeated in duplicate with similar results.

**Figure 9:**
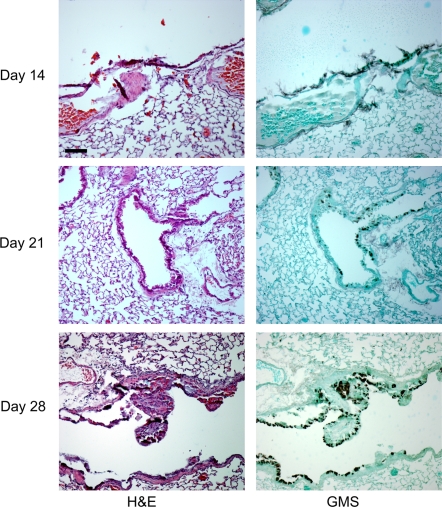
Representative histopathology of CD-1 mouse model SDW1 infected survivors. Hematoxylin and eosin (H&E) or Gommori's methenamine silver (GMS) stains at 100-fold magnification. No sign of inflammation or fungal burden was observed in any surviving animal on day +14, +21 and +28 of the infection. This result indicates that in this murine model, the immune system is capable of clearing the fungal infection in the absence of SrbA. Bar = 100 µm.

Next, we sought to determine whether SrbA was required for *A. fumigatus* virulence. To answer this important question, we utilized two distinct murine models of IPA. In the first model, outbred CD1 neutropenic mice infected with SDW1 displayed no symptoms associated with IPA (Figure 8A). This was in contrast to mice infected with the wild type CEA10 and reconstituted strain SDW2 that displayed well described symptoms of *A. fumigatus* infection including hunched posture, ruffled fur, weight loss, and increased respiration. Consequently, a significant difference in mortality was observed between the mice infected with SDW1 and mice infected with either SDW2 or CEA1O (P = 0.0002). Indeed, in this murine model, the SDW1 strain was completely avirulent (Figure 8A). We next asked the question whether mice infected with SDW1 were able to clear the infection. After 28 days, SDW1 infected mice displayed no visible or microscopic signs of infection. In particular, at days 14, 21, and 28 lung homogenates were taken from SDW1 infected mice and with the exception of one mouse, no fungal colonies were recoverable indicating that the mice had cleared the infection. Histopathological analyses of mice on days 14, 21 and 28 in this neutropenic model also confirmed the lack of fungal persistence and inflammation in mice infected with SDW1 (Figure 9).

**Figure 10:**
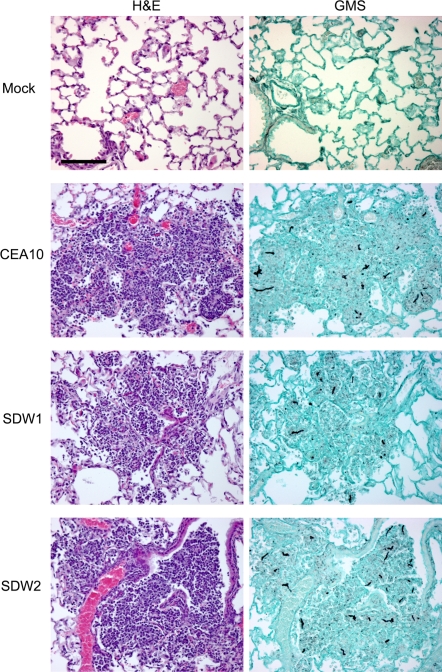
Histopathology of X-CGD mouse model 24 hours after infection. Mock = 0.01% Tween inoculated, WT = CEA10, SDW1 = *ΔsrbA*, SDW2 = *ΔsrbA+srbA*. Mice were inoculated with 1×10^6^ conidia intratracheally, euthanized on day +1 after inoculation, lungs removed, fixed in formaldehyde, and stained with hematoxylin and eosin (H&E) or Gommori's methenamine silver (GMS) stain. On day 1 no difference in size and state of lesions could be observed in the infected mice. GMS staining revealed that fungal colonization and germination is observed in all infected animals but not the mock control. This result indicates that SDW1 conidia are viable *in vivo* during the early stages of infection. Bar = 100 µm.

**Figure 11:**
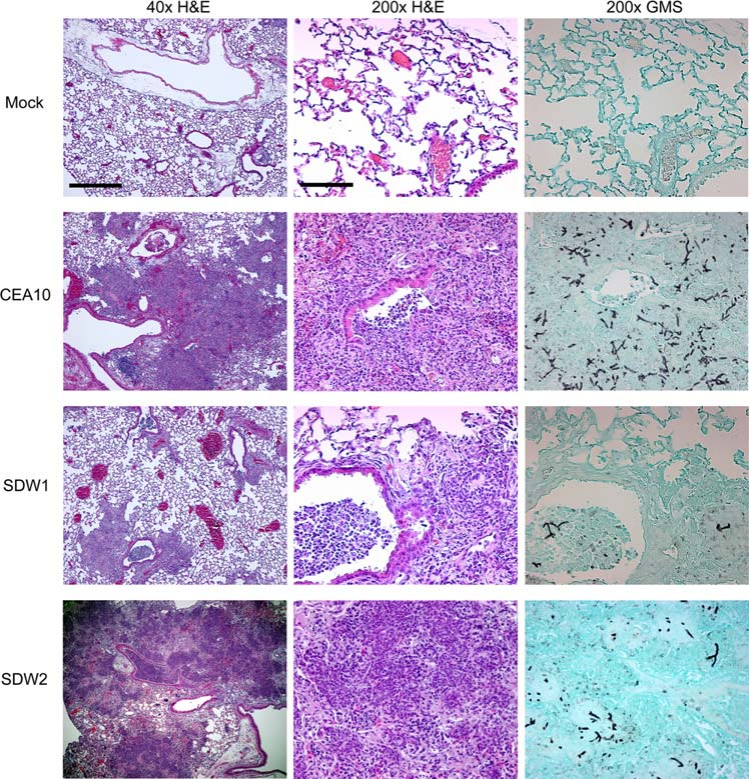
Histopathology of X-CGD mouse model day 4 after infection. Mock = 0.01% Tween inoculated, WT = CEA10, SDW1 = *ΔsrbA*, SDW2 = *ΔsrbA+srbA*. Mice were inoculated with 1×10^6^ conidia intratracheally, euthanized on day +4 after inoculation, lungs removed, fixed in formaldehyde, and stained with hematoxylin and eosin (H&E) or Gommori's methenamine silver (GMS) stain. Significant inflammation, necrosis, and an influx of immune effector cells (primarily neutrophils) is observed on day +4 in all infected animals but not the mock control. However, lesions are more localized and not as extensive in mice infected with SDW1. Open alveoli and more localized inflammation are clearly observed in mice infected with SDW1. Interestingly, GMS staining revealed that fungal growth is less extensive in SDW1 as well. This result indicates that as the infection progresses, SDW1 is incapable of continued hyphal growth despite the absence of NADPH oxidase in this murine model. Bar = 500 µm for 40×; Bar = 100 µm for 200×.

**Figure 12:**
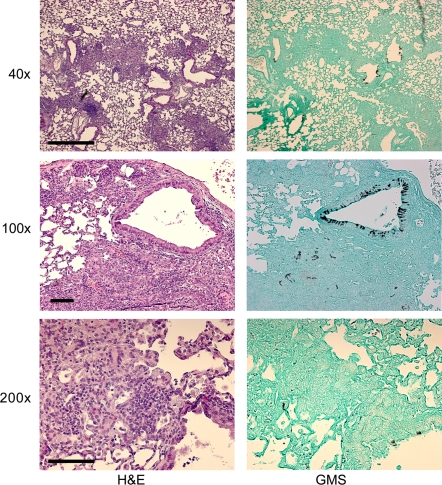
Representative histopathology of X-CGD mouse model SDW1 infected survivors. Hematoxylin and eosin (H&E) or Gommori's methenamine silver (GMS) stains. Resolution of inflammation and necrosis is observed in all surviving animals on day +14 of the infection. However, lesions are still apparent as is common in these mice, but necrosis and debris is significantly reduced. Fungal tissue remains evident on GMS stains indicating that despite surviving the infection, these mice have not entirely cleared the fungal infection. This result confirms the importance of a functional NADPH oxidase in resistance to *Aspergillus* infections, and suggests that increased hypoxia prevents proliferation of fungal tissue in the absence of SrbA. Bar = 500 µm for 40×; Bars = 100 µm for 100× and 200×.

Next, we examined the virulence of SDW1 in a murine model of X-linked chronic granulomatous disease (X-CGD) utilizing gp91*^phox−/−^* mice. These mice are deficient in NADPH oxidase activity and display hyper-susceptibility to *Aspergillus* species without the need for immunosuppression with chemotherapeautic agents [45],[46]. Similar to the neutropenic mouse model, X-CGD mice infected with strain SDW1 had significant differences in survival compared with mice infected with wild type and reconstituted strains (Figure 8B) (P = 0.005). Unlike the neutropenic mouse model, these mice all displayed symptoms of IPA during the preliminary stages of infection. These symptoms, likely due to the large inflammatory response characteristic of these mice when exposed to fungal antigens, included ruffled fur, hunched posture, and lethargic movement as early as 24 hours post-infection. However, only one mouse infected with SDW1 succumbed to the infection. In a repeat experiment, 3 additional X-CGD mice infected with SDW1 also succumbed on day 4 to the infection. Most likely, this was due to the hyper-inflammatory response that occurs in X-CGD mice and not death due to invasive fungal growth. Regardless, the majority of X-CGD mice infected with SDW1 survived the infection and displayed no symptoms of IPA by day 14. Histopathological analyses of these mice displayed standard pathological findings associated with *Aspergillus* infections in X-CGD mice including the development of granulomatous like lesions, massive influx of inflammatory cells (primarily neutrophils) to sites of infection, subsequent peribronchiolar and alveolar inflammation, and substantial fungal growth in silver stained tissue (Figures 10 and 11). On day 1 of the infection, fungal germination and growth is observed in mice infected respectively with all 3 strains of the fungus. This observation confirms the viability of SDW1 conidia *in vivo* (Figure 10). Semi-quantitative assessment of the percent of the lung affected by the infection, measured by inflammation and necrosis, of mice infected with the 3 strains respectively revealed no difference at this early time point (CEA10 = 1.3±0.5, SDW1 = 1.3±0.5, SDW2 = 1±0.0). Histopathology on day 4 of the infection, however, revealed extensive growth and proliferation of the wild type and reconstituted SDW2 strain, but minimal fungal growth and proliferation in mice infected with the SrbA null mutant SDW1 (Figure 11). Semi-quantitative assessment of the inflammation and necrosis observed in the lungs of mice infected with the 3 strains respectively at this time point revealed significant differences in the percent of the lung affected by the infection (CEA10 = 3.3±0.5, SDW1 = 2.3±0.5, SDW2 = 3.8±0.5). Lung homogenates from these mice also revealed that viable SDW1 fungus was recoverable from these mice at this time point. This data is consistent with the observed *in vitro* phenotype of the SDW1 strain in hypoxia. Histopathological analysis of SDW1 infected survivors in this model revealed persistence of granuloma like structures and fungal tissue (Figure 12). Lung homogenates from these animals revealed that the observed fungal tissue was still viable. These results indicate that despite normal growth rates *in vitro* in normoxic conditions, the SDW1 strain is severely attenuated in its ability to cause lethal disease in two distinct murine models of IPA.

### SrbA is not required for oxidative stress resistance and resistance to macrophage killing

**Figure 13:**
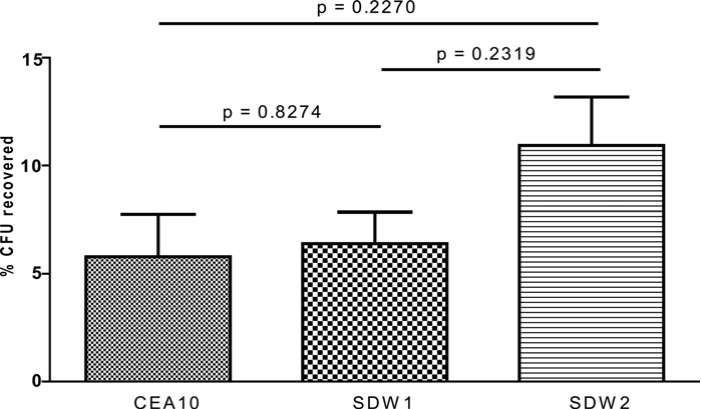
Loss of SrbA does not affect susceptibility to conidia killing by RAW264.7 cells. RAW264.7 cells (macrophages) were infected with a total of 1.25×10^6^ freshly harvested *A. fumigatus* conidia of strains CEA10, SDW1, and SDW2 to obtain a conidia∶macrophage ratio of 5∶1. Conidia and macrophages were incubated together for 6 hours. After 6 hours, conidia were collected from the macrophages and plated onto glucose minimal media. Shown is the percent of recovered conidia after 6 hours incubation of two biological replicates. No significant difference in conidia killing was observed between CEA10, SDW1, and SDW2 (P>0.01).

One possible mechanism that could explain the virulence defect of strain SDW1 is an increased susceptibility to oxidative stress as suggested by transcriptional profiling and altered conidia morphology. We examined the growth of CEA10, SDW1, and SDW2 in the presence of 1 mM and 2.5 mM hydrogen peroxide on glucose minimal media. After 48 hours, we observed no detectable difference in growth morphology or colony diameter. In addition, we next examined the ability of RAW264.7 macrophage-like cells to kill SDW1 conidia (Figure 13). As presented in figure 13, no significant difference in conidia killing was observed between CEA10, SDW1, and SDW2 (P>0.01). We conclude that increased susceptibility to oxidative stress and macrophage killing is not responsible for the virulence defect observed in the absence of SrbA.

## Discussion

In this manuscript we present the first characterization of a SREBP in a filamentous fungus. In the yeasts *S. pombe* and *C. neoformans*, SREBP homologs are crucial for sterol biosynthesis, survival under hypoxic conditions, resistance to azole antifungal agents, and fungal virulence [25],[32],[33]. Our results confirm that some roles of SREBPs in filamentous fungi are conserved with yeast including, the response to hypoxia, sterol biosynthesis, and susceptibility to the azole class of antifungal drugs. However, our results suggest additional functions of SREBPs in filamentous fungi, most importantly a role in maintenance of cell polarity.

Similarities and differences between SrbA in *A. fumigatus* and Sre1 in the yeast *S. pombe* and *C. neoformans* were apparent from transcriptional profiles comparing the SREBP null mutants to their respective wild type strains in response to hypoxia. Unlike *C. neoformans*, we did not observe SrbA dependent genes involved in iron or copper uptake in *A. fumigatus* [32]. This may, however, be a reflection of the experimental conditions that did not place iron stress on the fungus in these experiments. Similar to *C. neoformans* and *S. pombe*, we observed SrbA dependent genes involved in ergosterol biosynthesis including ERG25, ERG24, and ERG3 [31]–[33]. This result suggests that regulation of ergosterol biosynthesis is a conserved function of SREBPs in fungi. In *A. fumigatus*, the SrbA dependent regulation of ERG25 seems to be of particular significance as sterol profiles of the SrbA mutant indicated an accumulation of C-4 methyl sterols suggesting a block in ERG25 function. The effects of decreased ERG3 and ERG24 transcription in the SrbA null mutant is less clear. The accumulation of pathway intermediates may subsequently affect the expression of these genes, and thus, their regulation by SrbA may be indirect. Moreover, *A. fumigatus* is predicted to have 3 possible orthologs of ERG3 and two of ERG24, which likely indicates a complex regulatory mechanism for ergosterol biosynthesis in *A. fumigatus* that is mediated in part by SrbA under specific conditions such as hypoxia [47],[48]. Indeed, single mutants of *erg3* genes result in no difference in their sterol profiles compared with wild type strains [49].

Other differences with yeast in the transcriptional profile of the SREBP mutant in *A. fumigatus* suggest important roles for SrbA in filamentous fungal biology. For example, a non-ribosomal peptide synthetase, NRPS1 (or *pes1*), had the highest change in expression between wild type and the SrbA null mutant [43],[44]. This NRPS has been observed to mediate resistance to oxidative stress in *A. fumigatus* and displayed an attenuated virulence phenotype in a *Galleria mellonella* (wax moth) model of aspergillosis depending on inoculum dose [44]. NRPSs are not generally found in most yeast and are particularly abundant in filamentous fungi. Thus, this result suggests that the uncharacterized peptide produced by this NRPS may possibly be involved in hypoxia adaptation as regulated by SrbA in filamentous fungi. Interestingly, we did not observe any increased susceptibility to oxidative stress in the SrbA null mutant. Overall, however, unlike *C. neoformans* and *S. pombe*, we did not observe any genes with an annotation that would clearly point to a role in allowing *Aspergillus* to adapt to hypoxia. This result further illustrates that mechanisms of hypoxia adaptation are almost certainly different in molds than yeast.

Our examination of the SrbA null mutant colony morphology subsequently revealed abnormal branching at the hyphal tips in normoxia and an inability of hyphal growth in hypoxia. Further examination of the mutant with TEM suggested altered vesicle translocation or formation in the hyphae. It is unclear whether these electron dense objects, which we hypothesize are vesicles, comprise the actual Spitzenkörper. At the apex of hyphae in filamentous fungi, the Spitzenkörper is an accumulation of vesicles that is critical for growth directionality [50],[51]. Interestingly, Takeshita et al. (2008) recently observed that localization of key deposition proteins involved in polarized growth at the hyphal tip requires apical sterol-rich membranes [52]. Thus, we hypothesize that the altered hyphal morphology and excessive branching at the tips observed in the SrbA null mutant is due to the alteration in sterol composition of the sterol-rich microdomains in the membrane that are critical for localization of important vesicles and landmark proteins [53]. The alteration in sterol content may cause improper sorting of the vesicles to the apex of the hyphal tip. It is also likely then that the inability of the mutant to grow in hypoxia is related to the perturbation in sterol biosynthesis, a highly oxygen dependent pathway reported to require at least 22 molecules of oxygen.

We could not rescue the SrbA phenotype in hypoxia with addition of ergosterol or lanosterol (data not shown). Nor did exogenous addition of these sterols alter growth of the wild type strain in hypoxia as is the case for *S. cerevisiae*, which requires exogenous sterols for anaerobic growth. These results may suggest that *A. fumigatus* does not import exogenous sterols in hypoxic conditions, that SrbA may be in part responsible for exogenous sterol uptake, or that the defect is not due to loss of ergosterol or lanosterol. We feel that the latter explanation is most likely as *A. fumigatus* has been observed to take up and utilize exogenous cholesterol [54]. We observed that the *A. fumigatus* SrbA null mutant produced substantial levels of ergosterol even in the absence of SrbA. Thus, even though the ergosterol biosynthesis pathway appears blocked at ERG25 in the SrbA mutant, alternative mechanisms exist for *A. fumigatus* to produce ergosterol in the absence of SrbA and presumably ERG25 activity. This finding is consistent with a recent report which suggested that *A. fumigatus* likely possess at least three alternative pathways for ergosterol biosynthesis [48]. Also, an analysis of the *A. fumigatus* genome sequence revealed that *A. fumigatus* contains duplicate and even triplicate copies of many of the ergosterol biosynthesis genes [47],[55]. Thus, it appears that *A. fumigatus* contains complex regulatory mechanisms, of which SrbA is a part, for the production of ergosterol that remain to be elucidated.

Based on our current knowledge of the pathophysiology of IPA, the *in vitro* phenotypes observed in the SrbA mutant would not predict a role for this protein in *A. fumigatus* virulence. However, the SrbA null mutant was virtually avirulent in two distinct murine models of IPA despite a normal growth rate of the fungus in standard laboratory conditions. Consequently, we believe two possible explanations exist for the observed avirulent phenotype of the SrbA null mutant. First, and we believe most likely, the inability of the SrbA null mutant to grow in hypoxia prevents invasive disease from being established. Once hypoxia is generated during *Aspergillus* infection, the mutant simply can no longer grow and proliferate, allowing what immune effector cells that remain functional the ability to ultimately clear the infection. An alternative hypothesis is that the altered hyphal morphology and excessive branching observed in the SrbA mutant in normoxic conditions results in a strain incapable of invasive growth or a strain more susceptible to clearance by the immune system. To examine these alternatives, we employed the use of two distinct murine models of IPA.

We first examined the SrbA mutant virulence phenotype in a persistently neutropenic mouse model characterized by the use of high doses of cyclophosphamide and Kenalog [42]. Currently, it is unclear what specific components of the immune system are affected in this model, but it is clear that differences in the immunosuppression regimen can significantly affect the outcome of infection [56],[57]. In this model, significant inflammation and tissue necrosis is observed in histopathological examinations. We hypothesize that these sites of infection and inflammation in this model are hypoxic. Thus, we believe that *A. fumigatus* must overcome significant hypoxia during pulmonary infections, and the inability of the SrbA null mutant to adapt to hypoxic conditions results in rapid cessation of invasive growth and a lack of lethal disease. Our histopathological findings with the SrbA mutant strain revealed fungal growth in this model early in the infection. However, by day 14, we were unable to recover viable colonies from mice infected with the SrbA null mutant strain. Indeed, by day 14 of the infection, little evidence of inflammation or fungal burden was evident in mice infected with the SrbA null mutant. These two results suggest that growth of the fungus was halted and what immune effector cells present in the immunosuppressed mice were able to clear the infection. Furthermore, our *in vitro* experiments revealed that the growth defect of the SrbA mutant in hypoxia was not fungicidal but fungistatic. Thus, if growth simply were halted in the animals without immune system clearance, we would have expected to recover viable fungal colonies from the infected mice.

To further examine the apparent virulence defect of the SrbA null mutant, we utilized a mouse strain highly susceptible to *Aspergillus* infections, the X-CGD gp91*^phox−/−^* mice [45],[46]. These mice exhibit a hyper-inflammatory response when exposed to *A. fumigatus* and other *Aspergillus* species. We chose this particular animal model for our experiments given the very specific defect in NADPH oxidase function in these mice, and with the hypothesis that the hyper-inflammatory response would generate significant hypoxia in the lung. Given the extreme susceptibility of these mice to *A. fumigatus*, we hypothesized that if the SrbA null mutant could grow and persist *in vivo*, even at a reduced rate, we should observe significant mortality in these mice. However, in contrast, we observed limited mortality in these mice when inoculated with the SrbA null mutant, strongly suggesting that the mutant simply cannot grow effectively *in vivo* to cause invasive disease. Unlike the neutropenic mouse model, extensive signs of chronic inflammation remained evident in the X-CGD mice post-day 14, consistent with previously reported results in these animals [45]. Furthermore, unlike the neutropenic mice, we could detect the persistence of viable SDW1 in the lungs of these surviving mice out to day 14.

Consequently, we conclude that these observations strongly suggest that the inability of the SrbA null mutant to grow in hypoxic microenvironments is primarily responsible for the avirulent phenotype of the mutant. Though the altered cell polarity of the SrbA mutant may contribute to the virulence defect, the fact that SrbA null mutant displayed normal growth rates *in vitro* in standard laboratory growth conditions suggests to us that the altered cell polarity did not significantly affect fungal growth. Furthermore, we also have examined the susceptibility of the SrbA null mutant conidia to macrophage (RAW264.7 cells) killing and found no difference with the wild type strain. In addition, the SrbA mutant did not display increased sensitivity to hydrogen peroxide. Taken together, we feel these observations strongly suggest that the virulence defect in the SrbA null mutant is due to its inability to grow in hypoxia.

An additional observation of clinical significance was the finding that SrbA mediates resistance to the azole class of antifungal drugs. Interestingly, loss of SrbA resulted in a strain of *A. fumigatus* highly susceptible to fluconazole, an azole that normally has minimal activity against *A. fumigatus* [58],[59]. The mechanism(s) behind this result are currently not known. Transcriptional profiling of the SrbA mutant revealed numerous transporters possibly regulated by SrbA. Thus, the mechanism behind the increased azole susceptibility may be due to loss of transcription in specific transporters in the SrbA mutant. This hypothesis is currently being tested in our laboratory. Second, a relationship between mitochondria function, sterol homeostasis, and azole drug resistance has been observed in the yeast *S. cerevisiae* and *Candida glabrata* [60],[61]. Thus, the altered accumulation of sterol intermediates in the SrbA mutant may alter the resulting interaction with fluconazole and mitochondria. With a similar increase in susceptibility to azoles in the SREBP mutant in *C. neoformans*, it seems clear that further study of the SREBP pathway and azole drug resistance in pathogenic fungi is highly warranted. Identification of ways to inhibit this pathway *in vivo* may increase the efficacy of current azole antifungal agents [32],[33]. Thus, further studies are needed to dissect this important pathway in yeast and molds to identify conserved targets that may be harnessed to treat patients with invasive mycoses.

Finally, in this study, we did not focus on elucidating the molecular mechanism behind SrbA regulation and activation in molds. However, several observations from our studies hint at possible mechanisms. First, we identified SrbA in a transcriptional profiling screen of *A. fumigatus* in response to hypoxia (induced >5 fold). This suggests that SrbA may be transcriptionally regulated in molds. However, HIF1 in humans also responds transcriptionally to hypoxia, but its activity is primarily post-translationally regulated [62],[63]. In the yeast *S. pombe* and *C. neoformans*, it seems clear that Sre1 is regulated post-translationally in response to sterol biosynthesis perturbation that occurs in low oxygen environments. Indeed, Hughes et al. (2007) have identified 4-methyl sterols as the primary activating agent of Sre1 in *S. pombe* [64]. Thus, our finding that the SrbA null mutant in *A. fumigatus* accumulates 4-methyl sterols may also suggest that these sterols are the trigger for SrbA activation in *A. fumigatus*.

While many of the phenotypes we observed in the SrbA mutant in *A. fumigatus* may suggest that SrbA is regulated in a similar manner as Sre1 in yeast, our results may also suggest an alternative model in molds. First, despite extensive bioinformatic analyses, we were unable to identify a clear homolog of the sterol cleavage activating protein (SCAP). SCAP is highly conserved in yeast, mammals, and insects and thus it is surprising that bioinformatic searches were unable to identify a clear homolog in any filamentous fungi with genome sequences available. However, some candidates with minimal sequence similarity are being pursued in our laboratory. Second, the observation that sterol biosynthesis was altered in normoxia, likely resulting in altered cell polarity, suggests that in molds, SrbA plays a significant role in the biology of filamentous fungi in normoxic conditions. Third, though sequence identity was extremely low, generation of null mutants in putative site-1 (S1P) and site-2 (S2P) protease homologs in *A. fumigatus* did not demonstrate expected defects in hypoxic growth (Willger and Cramer, unpublished data). Additional proteases remain to be explored. We could, however, identify a clear Insig1 homolog, which we have named InsA. In mammals, Insig is a key regulator of SREBP function where it binds to SCAP and prevents SREBP cleavage in the presence of sterols by maintaining the SREBP-SCAP complex in the endoplasmic reticulum membrane [65],[66]. We are currently characterizing a possible role for InsA in SREBP signalling in filamentous fungi. Interestingly, *C. neoformans* lacks an apparent Insig homolog and the Insig homolog in *S. pombe* does not appear to be required for regulation of SREBP signalling [25],[32]. Taken together, these results suggest that while aspects of SrbA signalling in filamentous fungi may be conserved in yeast and mammals, it is likely that significant differences exist in molds that remain to be elucidated. What is clear, however, is that SREBPs play critical roles in the biology of fungi that have important implications for fungal virulence and how we manage and treat invasive fungal infections. Future studies on this pathway in *A. fumigatus* are likely to yield important insights into sterol metabolism, hypoxia adaptation, fungal growth, and mechanisms of azole drug resistance.

## Materials and Methods

### Strains and media

*A. fumigatus* strain CEA17 (a gift from Dr. J.P. Latgé, Institut Pasteur) was used to generate the *srbA* null mutant strain, SDW1 (*ΔsrbA::A. parasiticus pyrG pyrG1*). *A. fumigatus* strain CEA17 is a uracil-auxotrophic (*pyrG1*) mutant of *A. fumigatus* strain CEA10 [67],[68]. In this study we used CEA10 (gift from Dr. Thomas Patterson, University of Texas- San Antonio Health Sciences Center) as the wild type, SDW1, and an ectopic complemented control strain SDW2 (*Δsrb::A. parasiticus pyrG*+*srbA*). All strains were stored as frozen stocks with 50% glycerol at −80°C. The strains were routinely grown in glucose minimal medium (GMM) with appropriate supplements as previously described [69] at 37°C. To prepare solid media 1.5% agar was added before autoclaving.

### Strain construction

Generation of a *srbA* null mutant in *A. fumigatus* strain CEA17 was accomplished by replacing an ∼2.2-kb internal fragment of the *srbA* coding region (∼3.0 kb; GenBank accession no. XM_744169) with *A. parasiticus pyrG*. The replacement construct was generated by cloning a sequence homologous to the *srbA* locus into plasmid pJW24 (donated by Dr. Nancy Keller, University of Wisconsin—Madison). Homologous sequences, each ∼1 kb in length and 5′ and 3′ of the *srbA* coding sequence, were cloned to flank *A. parasiticus pyrG* in pJW24. The resulting plasmid, pSRBAKO, was used as a template to amplify the ∼5.1-kb disruption construct for use in fungal transformation. To complement the *ΔsrbA* strain SDW1 the *srbA* gene was amplified using genomic DNA of CEA10 as template and the primers 5′SrbAKOLF and 3′SrbAKORF. The ∼5.9-kb PCR product was used in a fungal transformation and selection was for colonies able to grow under hypoxic conditions. The primers utilized in vector construction are presented in Table S1.

Generation of fungal protoplasts and polyethylene glycol-mediated transformation of *A. fumigatus* were performed as previously described [70]. Briefly, 10 µg of the *srbA*KO PCR-generated replacement construct was incubated on ice for 50 min with 1×10^7^ fungal protoplasts in a total volume of 100 µl. Transformants were initially screened by PCR to identify potential homologous recombination events at the *srbA* locus. PCR was performed with primers designed to amplify only the disrupted *srbA* locus (5′SrbAKOLF and 3′PyrGKOLF; 5′PyrGKORF and 3′SrbAKORF) (Table S1). Homologous recombination was confirmed by Southern analysis with the digoxigenin labeling system (Roche Molecular Biochemicals, Mannheim, Germany) as previously described [71]. To eliminate the chance of heterokaryons, each transformant was streaked with sterile toothpicks a minimum of two times to obtain colonies from single conidia.

### Hypoxic cultivation

Strains were grown on GMM plates at 37°C. Normoxic conditions were considered general atmospheric levels within the lab (∼21% O_2_). For hypoxic conditions a Hypoxia Incubation Chamber (MIC-101; Billups-Rothenberg, http://www.hypoxiaincubator.com) was used. The chamber was maintained at 37°C and kept at ∼1% oxygen level utilizing a gas mixture containing 1% O_2_, 5% CO_2_ and 94% N_2_. In addition, hypoxia experiments requiring shake-flask cultures were conducted in a Biospherix C-Chamber with O_2_ levels controlled by a PRO-Ox controller and CO_2_ levels controlled with PRO-CO_2_ controller (Biospherix, Lacona, NY). For these experiments, O_2_ set point was 1% and CO_2_ set point was 5%.

Colony growth was quantified as previously described [72]. Briefly, 5-µl aliquots containing 1×10^6^ conidia from freshly harvested GMM plates were placed in the center of GMM agar plates. Plates were then cultured under normoxic or hypoxic conditions. Diameters of three colonies per *A. fumigatus* strain and condition were measured once daily over a period of 4 days. The average change in colony diameter per 24 h of growth was calculated from three independent cultures. Conidia were harvested with 20 ml of sterile 0.01% Tween 80, filtered through two layers of sterile miracloth (EMD Biosciences, La Jolla, CA), and quantified.

### Isolation of total RNA

Conidia from freshly harvested GMM plates were inoculated in 5 ml GMM in a 6-well plate to a concentration of 1×10^7^/ml. Cultures were grown aerobically for 24 h. For normoxic growth, cultures were maintained in atmospheric conditions. For hypoxic growth, cultures were placed in the hypoxic chamber for 24 h. Fungal mats were flash frozen in liquid nitrogen and lyophilized prior to total RNA extraction using TRIsure Reagent (Bioline) according to the manufacturer's instructions. RNA was further purified using the RNeasy Mini Kit (Qiagen) and re-suspended in DEPC-treated water. RNA integrity was confirmed with an Agilent Technologies Bioanalyzer.

### Microarray-based transcriptional profiling

Total RNA was reverse transcribed by priming with oligo dT and utilizing aminoallyl-dUTP. The resultant cDNA was then coupled to Cy3- and Cy5-labeled probes (GE Healthcare), and hybridized to *Aspergillus fumigatus* version 3 microarrays from the pathogen functional resources center (PFGRC) as described in the TIGR standard operating procedures found at http://atarray.tigr.org. Labeled cDNA from wild type grown in hypoxic conditions was hybridized against cDNA from SDW1 grown in hypoxic conditions. Data for each strain represents six independent experiments and includes three dye swaps. Arrays were scanned on an Axon 4000B scanner with GenePix software at the Montana State University Functional Genomics Core facility (Axon Instruments). Array signals were bulk-normalized and filtered for flagged spots using MIDAS (available at http://www.tm4.org/midas.html). Data were log-transformed (base 2) and filtered for genes that contained data for at least three out of four arrays from each strain, and missing values were calculated through K-nearest neighbor algorithm using Significance Analysis of Microarrays (SAM) software [73] prior to statistical analysis by SAM. Statistically significant genes identified by SAM with 2-fold or greater changes in expression are listed in Table 1. A Delta cutoff in SAM that captured the maximum number of significant genes with a false discovery rate of zero was utilized. Microarray data has been deposited in the Gene Expression Omnibus (GEO) at the National Center for Biotechnology Information (NCBI) series accession number GSE12376.

### Susceptibility testing

E-test strips (AB Biodisk, N.J.) plastic strips impregnated with a gradient of Fluconazole, Voriconazole, Caspofungin, or Amphotericin B were used per manufacturers' instructions. Each strip was placed onto a RPMI-1640 (Sigma Aldrich) agar plate containing a lawn of conidia and growth inhibition was measured after 24 and 48 h by direct observation of the plates at 37°C. No difference in results was observed between 24 and 48 h.

### Sterol analyses

Sterols were extracted following published protocols [74]. Gas-chromatography-Mass spectrometry analyses were performed with a HP6890 GC coupled to a HP5973 mass selective detector. Electron impact MS (70 eV, scanning from 50 to 550amu, at 2.94 intervals/sec) was performed using the following conditions: HP-5 column (30 m×0.25 mm i.d., 0.25 mm film thickness), Helium as carrier gas (1 ml/min), detector temperature 180°C, column temperature 100°C to 300°C (100°C for 1 min, 7°C/min to 300°C then held for 15 min). All injections were run in splitless mode.

### Electron microscopy

Conidia and mycelia of wild type and SDW1 were examined by transmission electron microscopy (TEM). Conidia released in sterile water from 5-day-old GMM plates and mycelia grown in liquid GMM for two days were collected by centrifugation at 5000× g for 10 min. The conidial and mycelial pellets were coated with 0.8% agarose and fixed in modified Karnovsky's fixative containing 2% paraformaldehyde and 2% (v/v) glutaraldehyde in 0.05 M sodium cacodylate buffer (pH 7.2) overnight at 4°C. After washing three times with 0.05 M sodium cacodylate buffer (pH 7.2) for 10 min each, samples were post-fixed with 1% (w/v) osmium tetraoxide in the same buffer for 2 hours at 4°C. The post-fixative was removed by washing briefly twice with distilled water at room temperature and the samples were *en bloc* stained with 0.5% uranyl acetate overnight at 4°C. The samples were then dehydrated in a graded ethanol series, rinsed with propylene oxide, and embedded in Eppon resin (Fluka AG, Zürich, CH). Ultrathin sections cut from the Eppon-embedded material with ultramicrotome (MT-X, RMC, USA) were collected on carbon-coated grids, stained with 2% uranyl acetate for 3 min, and with Reynold's lead solution [75] for 3 min. Examination was conducted with a JEM-1010 (JEOL, Tokyo, Japan) electron microscope operating at 60 kV.

### Conidia Germination assay

For the conidia germination assay, *A. fumigatus* strains were grown in 25 ml GMM with 2% yeast extract. Cultures were inoculated with approximately 10^6^ conidia/ml. After 7 hours the germination rate was determined by counting a total of 100 spores and noting the number of germinated spores. Counting was repeated three times for each strain and the mean and standard deviation are reported.

### Murine virulence assays

In this study two different mouse models were used to assess the role of the transcription factor SrbA in fungal virulence. For the persistently neutropenic mouse model we used outbred CD1 (Charles River Laboratory, Raleigh, NC) male mice (26 to 28 g in size, 6–8 weeks old), which were housed six per cage and had access to food and water ad libitum. Mice were immunosuppressed with intraperitoneal (i.p.) injections of cyclophosphamide at 150 mg/kg 2 days prior to infection and with Kenalog injected subcutaneously (s.c.) at 40 mg/kg 1 days prior to infection. On day 3 post-infection (p.i.), repeat injections were given with cyclophosphamide (150 mg/kg i.p.) and on day 6 p.i. with Kenalog (40 mg/kg s.c.). Twelve mice per *A. fumigatus* strain (CEA10, *srbA*-deficient mutant SDW1, or the reconstituted strain SDW2) were infected intranasally. For an alternative mouse model, we used breeder mice with a null allele corresponding to the X-linked gp91*^phox^* component of NADPH oxidase (B6.129S6-*Cyb^btm1Din^*). Breeding pairs of these mice were obtained from the Jackson Laboratory (Bar Harbor, Maine) and reared in the Animal Resource Center at Montana State University. All animals were kept in specific-pathogen-free housing, and all manipulations were approved by the institutional internal review board (IACUC). To avoid exposing gp91*^phox−/−^* mice to bacterial infections, they were housed in microisolator cages in an environment of filtered air and given autoclaved food ad libitum and prophylactic treatment with sulfamethoxazole-trimethoprim in their sterile drinking water. The animals were used at 8 to 13 weeks of age. The mice were inoculated intratracheally following brief isoflurane inhalation, returned to their cages, and monitored at least twice daily.

Infection inoculum was prepared by growing the *A. fumigatus* isolates on GMM agar plates at 37°C for 3 days. Conidia were harvested by washing the plate surface with sterile phosphate-buffered saline-0.01% Tween 80. The resultant conidial suspension was adjusted to the desired concentration of 1×10^6^ conidia/40 µl by hemacytometer count. Mice were observed for survival for 14 days after *A. fumigatus* challenge. Any animals showing distress were immediately sacrificed and recorded as deaths within 24 h. Mock mice were included in all experiments and inoculated with sterile 0.01% Tween 80. Lungs from all mice sacrificed during the experiment were removed for fungal burden assessment and histopathology. Experiments were repeated in duplicates with similar results. Survival was plotted on a Kaplan-Meier curve and a log-rank test used to determine significance of pair-wise survival (two-tailed P<0.01). No mock infected animals perished in either murine model in all experiments.

### Histopathology

For histopathology studies, additional gp91*^phox−/−^* mice were infected as described above, and sacrificed at set time points of day 1 and day 4 after *A. fumigatus* challenge. When mice were sacrificed, lungs were removed on that day. Lung tissue was fixed in 10% phosphate-buffered formalin, embedded in paraffin, sectioned at 5 µm, and stained with hematoxylin and eosin (H&E) or Grocott methenamine silver (GMS) by using standard histological techniques. Microscopic examinations were performed on a Zeiss Axioscope 2-plus microscope and imaging system using Zeiss Axiovision version 4.4 software. Semi-quantitative analysis of inflammation and necrosis were scored on a scale of 1 to 5. The scale consisted of: 1 = 0 to 24% lung involvement, 2 = 25–49%, 3 = 50–74%, 4 = 75–99% 5 = 100%. H&E stained whole lungs from 4 mice infected with each respective strain were assessed to determine the percentage involvement and scored accordingly on days 1 and 4 of the infection in consultation with a pulmonary immunologist.

### Macrophage assays

Macrophage killing of conidia was measured by serial dilution as previously described with slight modifications [76]–[78]. Briefly, 2.5×10^5^ RAW264.7 cells in a volume of 500 ml were inoculated into 24 well tissue culture treated cell culture plates (Corning Incorporated, Corning, NY) in DMEM complete media and incubated overnight at 37°C, 5% CO_2_. A total of 1.25×10^6^ freshly harvested *A. fumigatus* conidia of the respective strains in DMEM complete media were inoculated into each well to give a conidia∶macrophage ratio of 5∶1. Co-incubation was performed at 37°C, 5% CO_2_ for 1 hour, after which media was removed and cells were gently washed with 1× phosphate buffered saline (PBS) to remove non-phagocytosed conidia. At this time point, conidia from each strain were harvested from macrophages in one well to establish the baseline number of conidia engulfed. DMEM complete media was added back to the non-harvested wells and incubation proceeded for an additional 5 hours. Lysis of macrophages was performed by treating the cells with 200 ml of a 0.5% SDS solution for 10 minutes followed by addition of 200 ml of 1× PBS. The percentage of colony forming units (CFU) from conidia∶macrophage co-incubations was determined relative to control conidia harvested at the one hour time point. Controls were performed by lysing macrophages as described above after phagocytosis of conidia for 1 hour and CFU counts were set to 100%. Experiments were performed with triplicate wells and repeated two times for each *A. fumigatus* strain.

### Oxidative stress assay

For the oxidative stress assay, the *A. fumigatus* strains were grown on GMM plates with and without H_2_O_2_. GMM plates with 1 and 2.5 mM H_2_O_2_ were prepared. Plates were inoculated with approximately 100,000 spores in 5 µl and incubated at 37°C. Sensitivity to oxidative stress was determined by comparing the colony radius of 2-day-old cultures on plates with H_2_O_2_. The assay was repeated three times for each concentration. Growth of each strain on each plate was visually examined.

### Statistical analysis

The software program Prism 5 (GraphPad, San Diego, Calif.) was used for all statistical tests of significance (to P values of ≤0.01). Normally, a two-sided t test was used to compare two groups of data, with Welch's correction being used if the groups had unequal variances. In cases in which a deviation from a normal distribution was suspected, a nonparametric test (Mann-Whitney test) was also applied. In those cases, we found that both the t test and Mann-Whitney test indicated the same results (i.e., both indicated significance or insignificance); however, typically one test gave a more conservative (larger, but still <0.01) P value. The P values we report are always the conservative values. Log-rank tests were utilized to determine significance of survival in animal studies.

## Supporting Information

Table S1 Primers Used in This Study
(0.03 MB DOC)
