# Supplementary material for: A Sterol-Regulatory Element Binding Protein Is Required for Cell Polarity, Hypoxia Adaptation, Azole Drug Resistance, and Virulence in Aspergillus fumigatus
Source: PLoS Pathog. 2008 Nov 7;4(11):e1000200. doi: 10.1371/journal.ppat.1000200 (PMC2572145; doi:10.1371/journal.ppat.1000200)
Supplement: Table S1 — Primers Used in This Study (0.03 MB DOC) [file ppat.1000200.s001.doc]

Table 1:

| **Primer** | **Sequence 5’ to 3’** | **Function** |
| --- | --- | --- |
| 5'EcoRILFSreA | AGG TCG ACT ACT GTT GAG TTG CTC AGG TGG CT | SreA disruption construct |
| 3'SalILFSreA | TCG AAT TCT TGG ATG GCG CCT GTT TAG CTT TG | SreA disruption construct |
| 5'XbaIRFSreA | GAT CTA GAT CAG ATG ATG GTG AAG ACG GTG CT | SreA disruption construct |
| 3'NotIRFSreA | GAG CGG CCG CAA CCA TAG ACC TTA CAG CCG CCA T | SreA disruption construct |
| 5'SreAKOLF | GAA GAA GTG GCG CAA ATC AAG GCT | Check homologous recombination left (5') |
| 3'PyrGKOLF | TAG GGT ACC TGT CCG CGC GGG G | Check homologous recombination left (5') |
| 5'PyrGKORF | TGG CGA CCA CAC CCG TCC TGT G | Check homologous recombination left (3') |
| 3'SreAKORF | TCT CGA TTG AGG TTC GAT GGG CAA | Check homologous recombination left (3') |
